# Supplementary figures and images for: The Effect of Isolated and Combined Application of Menthol and Carbohydrate Mouth Rinses on 40 km Time Trial Performance, Physiological and Perceptual Measures in the Heat
Source: Nutrients. 2021 Nov 29;13(12):4309. doi: 10.3390/nu13124309 (PMC8708984; doi:10.3390/nu13124309)

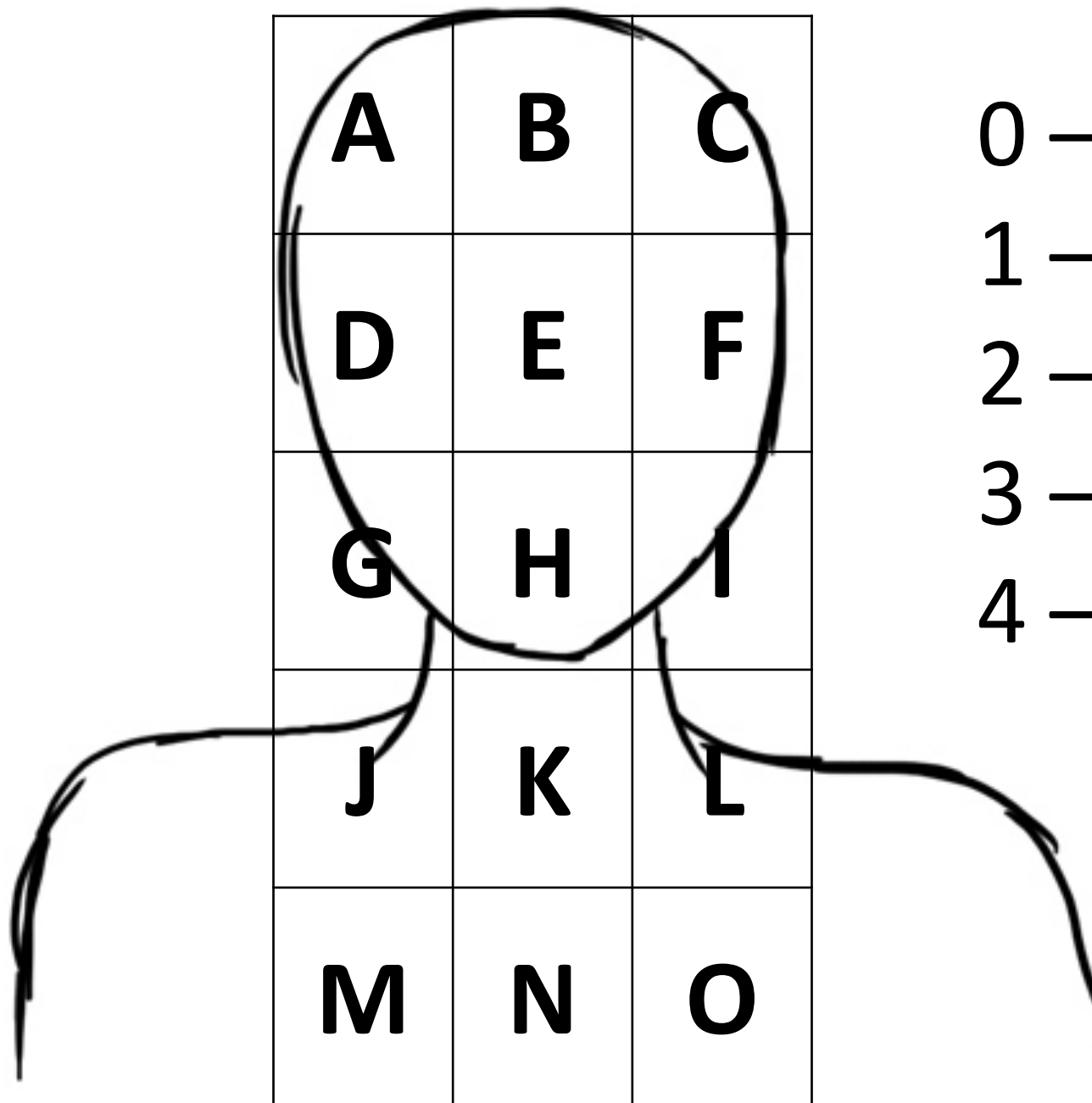

0 – None  
1 – Weak  
2 – Moderate  
3 – Strong  
4 – Very Strong

Supplement: Supplementary file 1 [file nutrients-13-04309-s001.zip › nutrients-1465139-supplementary.pdf]
